# A novel automated image analysis pipeline for quantifying morphological changes to the endoplasmic reticulum in cultured human cells

**DOI:** 10.1186/s12859-021-04334-x

**Published:** 2021-09-08

**Authors:** M. Elena Garcia-Pardo, Jeremy C. Simpson, Niamh C. O’Sullivan

**Affiliations:** 1 UCD School of Biomolecular and Biomedical Science, UCD Conway Institute, University College Dublin, Dublin 4, Ireland; 2 Cell Screening Laboratory, UCD School of Biology and Environmental Science, University College Dublin, Dublin 4, Ireland

**Keywords:** Endoplasmic reticulum morphology, ER function, High-content imaging, Automated image analysis

## Abstract

**Background:**

In mammalian cells the endoplasmic reticulum (ER) comprises a highly complex reticular morphology that is spread throughout the cytoplasm. This organelle is of particular interest to biologists, as its dysfunction is associated with numerous diseases, which often manifest themselves as changes to the structure and organisation of the reticular network. Due to its complex morphology, image analysis methods to quantitatively describe this organelle, and importantly any changes to it, are lacking.

**Results:**

In this work we detail a methodological approach that utilises automated high-content screening microscopy to capture images of cells fluorescently-labelled for various ER markers, followed by their quantitative analysis. We propose that two key metrics, namely the area of dense ER and the area of polygonal regions in between the reticular elements, together provide a basis for measuring the quantities of rough and smooth ER, respectively. We demonstrate that a number of different pharmacological perturbations to the ER can be quantitatively measured and compared in our automated image analysis pipeline. Furthermore, we show that this method can be implemented in both commercial and open-access image analysis software with comparable results.

**Conclusions:**

We propose that this method has the potential to be applied in the context of large-scale genetic and chemical perturbations to assess the organisation of the ER in adherent cell cultures.

**Supplementary Information:**

The online version contains supplementary material available at 10.1186/s12859-021-04334-x.

## Background

One significant challenge in the field of image analysis is the acquisition of images in a manner that permits readily quantifiable information about individual cells in a well or tissue. In this respect, automated analysis offers many advantages over manual assessment of images, as it is objective, reproducible, and significantly less labour intensive, enabling the analysis of large data sets in a fraction of the time [1]. However, the design of logical sequential steps of image analysis, so-called automated analysis pipelines, using commercial and open-access software (e.g. Harmony®, CellProfiler [2]), can be anything but straightforward depending on the phenotype being studied. In most image analysis pipelines, the nucleus is the primary organelle that is identified, as it is a large, spherical or ovoid, single-copy organelle per cell, from which basic geometrical features such as length (long-axis), width (short-axis), area, perimeter and circularity can readily be extracted. The next step is identification of the cell plasma membrane, which in turn provides information on the location of the cytoplasm. After these steps, most image analysis strategies then focus on identifying the organelles or subcellular structures of interest in the cytoplasm and implementing appropriate analysis and quantification routines [3]. Cellular organelles display a high degree of morphological diversity, and it is at this stage that image analysis pipelines diverge in order that the most appropriate methods to identify them can be applied. For example, although the Golgi complex is generally also a single organelle in mammalian cells, its appearance can be highly heterogeneous. Nevertheless, increasingly sophisticated image analysis routines are now able to report a variety of phenotypes seen in this organelle in response to various perturbations [4, 5]. Similarly, in recent years excellent progress has been made in automated detection and analysis of other organelles displaying punctate and tubular morphologies, such as endosomes [6]. Indeed even profiling of the Golgi and mitochondria in living cells allowing analysis of organelle fission and fusion events has also been shown to be possible [7, 8]. Despite this progress, the highly dynamic and morphologically complex endoplasmic reticulum (ER) network possesses a significant challenge to analyse using automated image analysis methods. As a result, there is a lack of available tools for the quantitative analysis of ER morphology, despite an increasing body of literature linking ER morphology with human disease [9, 10].

The ER is an extensive network of sheets and tubules extending from the nuclear envelope to the plasma membrane. Despite maintaining continuity of the luminal space, the ER is highly compartmentalised, comprising the nuclear envelope (NE), ribosome-studded sheets of “rough” ER (RER), a network of smooth ER (SER) tubules [11, 12], as well as specialised domains termed ER exit sites, which play a key role in the first steps of secretory transport [13]. Each compartment performs essential yet distinct cellular functions, such as protein synthesis and folding at the RER, and calcium storage, lipid metabolism, and mitochondrial division at the SER [14]. ER compartments also differ structurally. The RER comprises two parallel lipid bilayers that extend over many microns with little curvature, which are sealed at curved rims leaving a 50 nm thick lumen. These sheets are usually stacked, connected by regions of twisted membranes and helical edges [15]. By contrast, the SER comprises tubules with diameters ranging from 25 to 90 nm which are stabilised by ER-shaping proteins that create positive curvature [16–18].

Within post-mitotic cells, the ER is arranged such that the RER localises proximal to the nucleus and the Golgi apparatus, while the SER tubular network occupies both the perinuclear region and extends towards the cell periphery [19, 20]. However, ER organisation is dynamic and can alter depending on the physiological conditions of the cell. For example, the ER undergoes a complete reorganisation during mitosis by the interconversion of sheets-to-tubules and disassembly of the NE [21, 22]. Altered availability of ER resident proteins, cytoskeletal proteins, or phospholipids can also shift the balance between ER tubules and ER sheets [19, 23–25]. Maintaining ER organisation is vital to preserve the essential functions carried out by the ER [11]. Altered ER morphology has been reported in the context of several pathological conditions, for example (1) during viral infection viruses co-opt host cell membranes for their replication and in many cases remodel the ER membrane through interactions with ER proteins creating so-called replication factories (RFs), which promote enrichment of cellular and viral components necessary for viral replication [26, 27]; (2) the presence of protein aggregates, the hallmark of many neurodegenerative diseases, is linked to alteration of ER organisation by increased presence of enlarged peripheral ER sheets [28, 29]; (3) defects in misfolded protein clearance by impaired ER-associated degradation, or accumulation of misfolded proteins in cystic fibrosis cells, both result in dilated ER elements [30, 31]; (4) the cytotoxic effects of several chemicals have been linked to changes on ER structure [32–37]; and (5) mutations in ER-shaping proteins, known to cause neurodegenerative disorders such as hereditary spastic paraplegias (HSPs), have been shown to disrupt ER organisation in both in vitro and in vivo models [24, 38–40].

Despite the clear importance of ER organisation in cellular function and dysfunction, image analysis tools for the reliable and reproducible quantification of ER conformations are extremely limited. Published reports largely rely on qualitative descriptions of changes in ER structures, while quantification of changes in ER organisation are scarce or absent in most model systems [26, 29, 41–43]. Recently a set of tools has been developed which enables the quantification of the ER network in plant cells [44]. Given that the ER is a single copy organelle with vital roles in cell health and disease, there is a pressing need to establish objective tools for the description of this intricate organelle in cultured mammalian cells that will permit researchers to detect changes in its organisation under different conditions. In this study, we describe the design of a high-content platform for automated analysis of ER organisation. We have used this platform to study the effect of genetic and chemical modifiers on the organisation of ER in adherent cell cultures, demonstrating its robustness and applicability for wider use in various cell-based assays.

## Results

### A high-content and high-resolution imaging strategy to visualise the endoplasmic reticulum

**Fig. 1:**
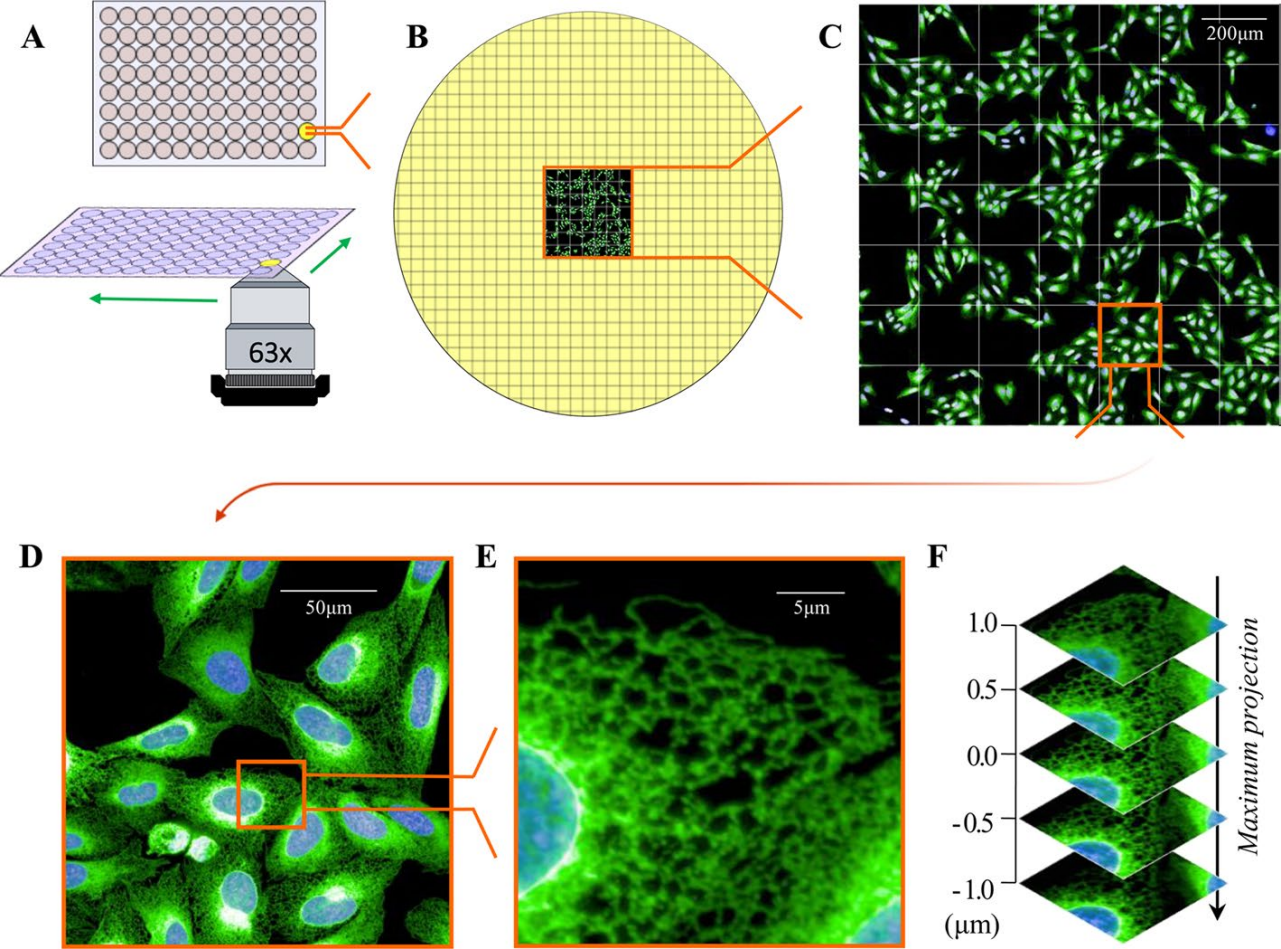
Automated high-content/high-resolution imaging workflow. **A** 96-well microplates were imaged using an automated spinning disk confocal microscope with a 63 × water immersion objective. **B** Total number of imaged fields (grid) present in a standard 0.32 cm^2^ well of a 96-well plate (yellow) including an imaged area of 7 × 7 fields. **C** Enlarged 7 × 7 imaged area containing U-2 OS cells stably expressing the ER marker Sec61β-mEmerald (green) and nuclei stained with Hoechst 33342 (blue). Scale bar = 200 μm. **D** Enlarged single imaging field. Scale bar = 50 μm. **E** Enlarged region of the imaged field showing the detail of the ER network obtained with the 63 × objective. Scale bar = 5 μm. **F** Images correspond to maximum intensity projection of 5 confocal planes interspaced at 0.5 μm intervals

Successful implementation of downstream image analysis relies upon the quality and consistency of the input images, and so the first step was to optimise our imaging protocol in such a way that it would allow for subsequent scale-up. Experiments were therefore designed in 96-well optically clear plates, compatible with a fully automated confocal high-content screening microscope fitted with a 63 ×/1.15 NA water-immersion objective (Fig. 1A). This experimental set-up was designed to ensure that all imaging conditions remained unaltered throughout the experiments, and was compatible with potential upscaling to large-scale genetic perturbation or chemical screening. One major bottleneck in high-content imaging combined with high-resolution microscopy is data accumulation [1]. Aiming to alleviate this problem, we sampled a central area of 1.53 mm^2^ in each well (Fig. 1B), ensuring that we captured a minimum of 50–100 cells per well, which is a number generally considered to be sufficient for accurate high-content analysis from images acquired with a 63 × objective lens (Fig. 1C). We next considered what would be the most relevant cell model with which to develop an image analysis pipeline for the ER. We initially selected U-2 OS cells, as these cells possess a large and flat morphology, with an extensive RER and SER network spread throughout their cytoplasm (Fig. 1D) [45]. Specifically, we used a U-2 OS cell line stably expressing the ER marker Sec61β-mEmerald [33]. This marker localises to both SER tubules and perinuclear RER, offers a strong signal to noise ratio (Fig. 1D), and its exogenous expression does not affect ER dynamics, as the microtubule binding site of the Sec61β protein is blocked by its fusion to the fluorescent protein mEmerald [46]. These U-2 OS cells were plated into wells of a 96-well plate and imaged the following day using a 63 ×/1.15 NA water-immersion objective, achieving an effective xy resolution of 0.28 μm, which enabled visualisation of peripheral SER tubules and dense ER structures (brighter) in the perinuclear region (Fig. 1E). Although U-2 OS cells are strongly adherent, and therefore their cytoplasm and organelles extend laterally over several microns in almost only 2-dimensions, they still have a small degree of depth that could potentially lead to under-detection of continuous ER tubules when only analysing a single optical slice. In order to overcome this issue, we determined that best practice was to image several z planes to span the depth of the periphery of the cells, and subsequently to use the maximum projection of these slices as the input image for the analysis (Fig. 1F). This approach also assists in eliminating out-of-focus artefacts.

**Fig. 2:**
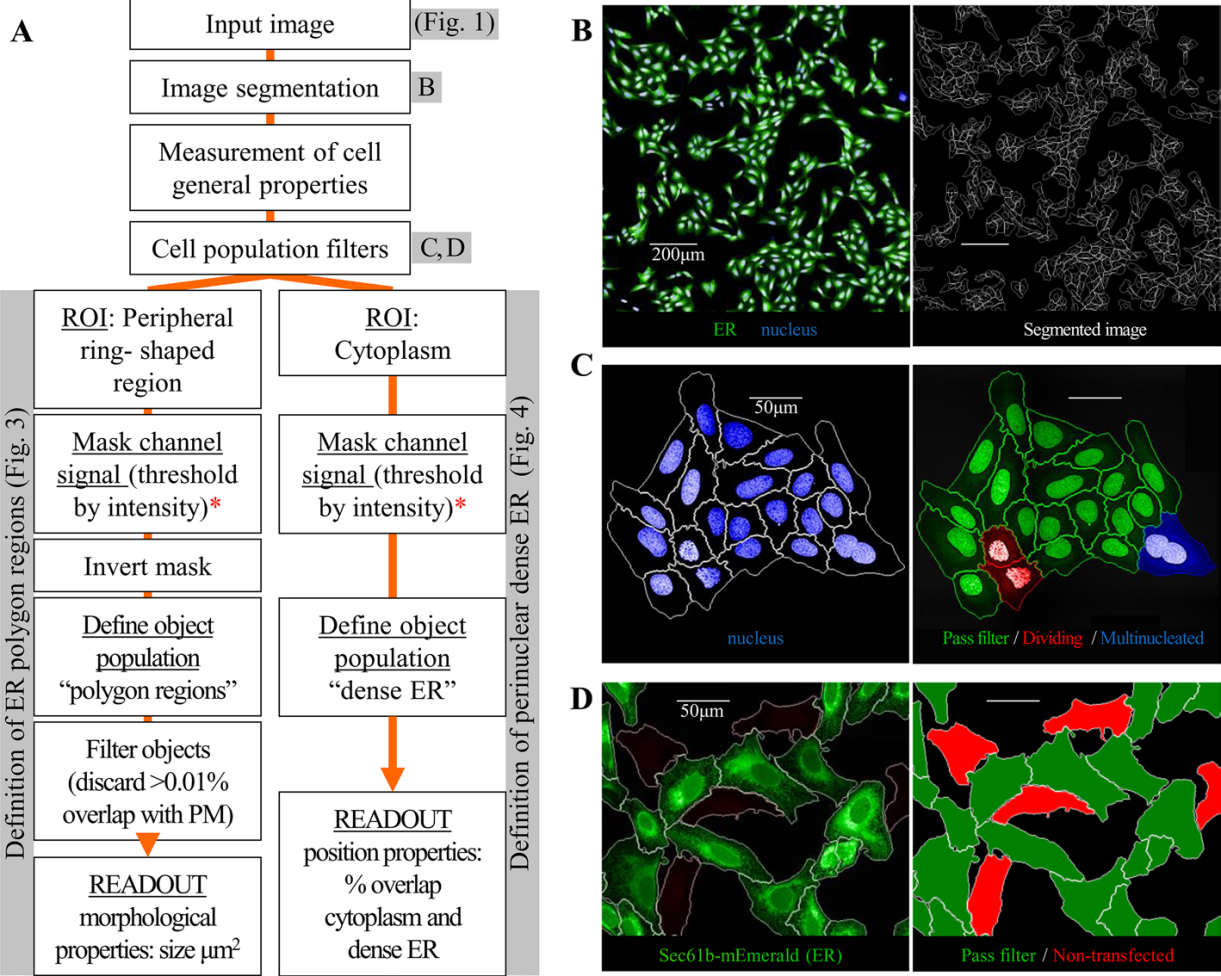
ER analysis pipeline workflow and preliminary steps. **A** Analysis pipeline workflow summary. ROI: Region of Interest. PM: plasma membrane **B** Example of segmentation of 7 × 7 imaging fields (left); identification of individual cells and cell regions: nucleus, PM and cytoplasm (right). Scale bars = 200 μm. **C** Filters based on nuclear morphological and intensity properties. (Left) Group of cells showing nuclei stained with Hoechst 33342. (Right) Filtered cells (green) and discarded cells: dividing cells (red) and multinucleated cells (blue). Scale bars = 50 μm. **D** Filters based on cytoplasmic ER marker (Sec61β-mEmerald) intensity. (Left) Group of cells showing ER labelled with Sec61β-mEmerald. (Right) Filtered cells (green) and discarded non-transfected cells (red). Scale bars = 50 μm

The analysis workflow developed is summarised in Fig. 2A and illustrates the principles followed. The first steps are standard in automated cell analysis and involve cell segmentation (Fig. 2B), and the filtering of the cell population based on nuclear properties to remove dividing and apoptotic cells (Fig. 2C). Given that we were working with a stably expressing cell line, we also included a filter step to remove cells that had either lost the exogenous DNA plasmid or were only weakly expressing it (Fig. 2D). We initially performed the analysis using Harmony® image analysis software, the bespoke analysis software for the automated microscope used to acquire the images, as this software allows easy stitching of multiple fields. However, in order to demonstrate that our analysis approach is not restricted to this software, we applied the same principles and recreated a similar analysis pipeline in CellProfiler [2] using maximum projection images of individual fields (Additional file 1: Fig. S1). Once all individual cells were segmented and filtered, we next built image analysis steps to quantify the ER organisation, specifically the area of SER polygons and the proportion of dense perinuclear ER (detailed below).

### Definition of SER polygon regions

**Fig. 3:**
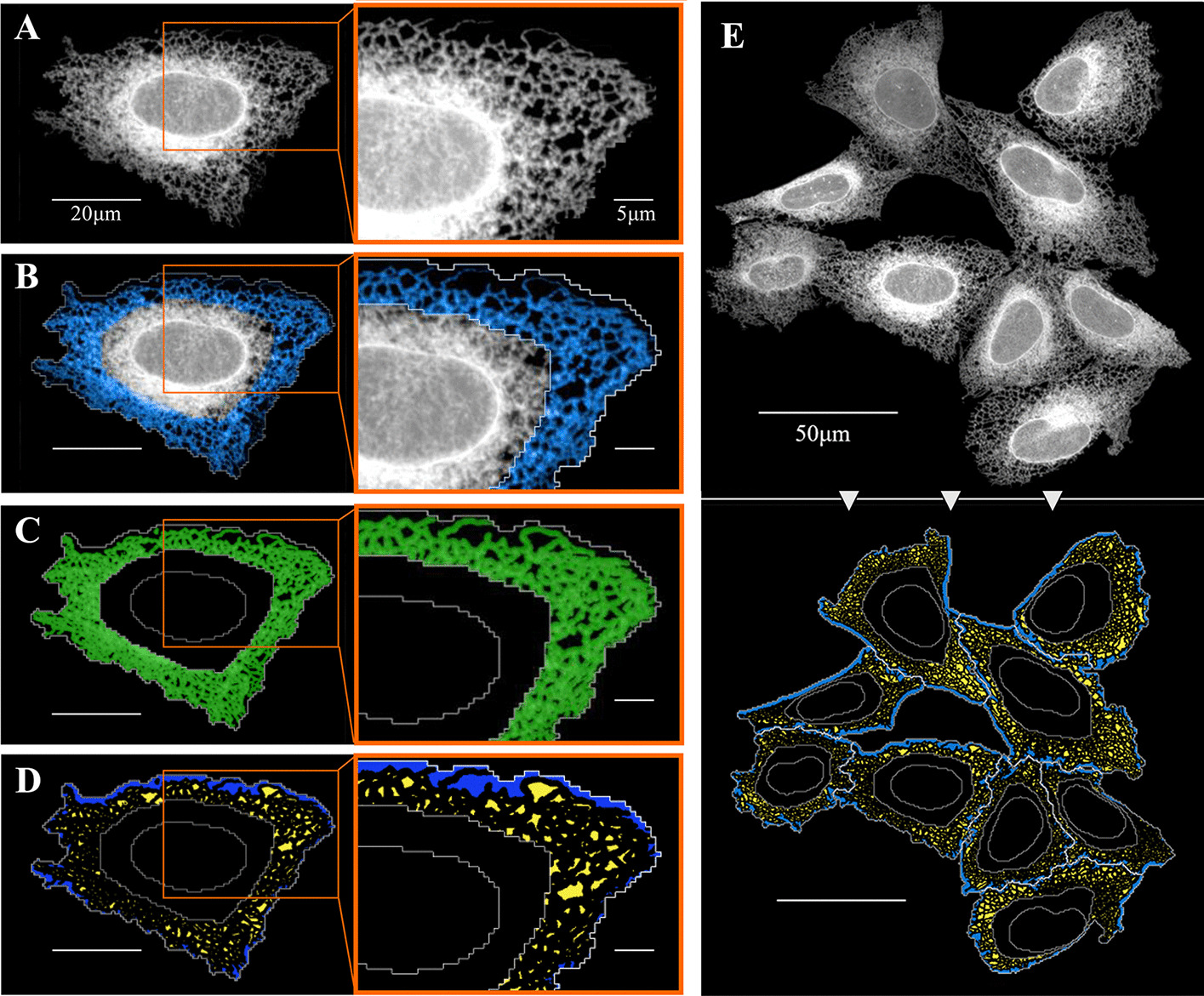
Definition of SER polygon regions. **A–D** Sequential key steps in the analysis shown on the same U-2 OS cell and enlarged region of the cell. Scale bars = 20 μm and 5 μm on the enlarged images. **A** U-2 OS cell expressing the ER marker Sec61β-mEmerald (white). **B** Definition of the peripheral ring-shaped region of the cytoplasm region of interest (ROI). **C** Masking of ER tubules (green) applying a threshold to the Sec61β-mEmerald signal within the ROI. **D** Inversion of ER mask resulting in detection of internal polygonal regions (yellow) and areas between the ER and the detected plasma membrane (blue). **E** Steps A and D shown in a group of cells. Scale bar = 50 μm

SER tubules form a network in a polygonal array, in which the “gaps” are termed polygonal regions [47]. SER tubules interconnect at three-way junctions, creating the nodes of the SER network. This morphology was clearly visible in our U-2 OS cells expressing Sec61β-mEmerald (Fig. 3A). A previous study reported an increase in the number of three-way junctions per membrane surface upon over-expression of the three-way junction resident protein Lunapark, giving rise to a densely branched network with smaller polygons [39]. Hypothesising that other perturbations to ER function may alter the number of three-way junctions and the density of the SER network, we explored the use of the polygonal region size as a readout of the density of the SER network in the cell periphery. The tubular SER network of polygons extend from the perinuclear RER, through the periphery of the cell to the plasma membrane [12, 48]. In order to quantify the area of these ER polygons, we first defined the cell periphery as a new region using the Harmony image analysis ‘building block’ termed “select cell region”, which performs radial calculations to shrink the cytoplasm, pushing the inner border (nuclear border) towards the cell perimeter depending on the distance between the two, resulting in a new a ring-shaped area which defines our region of interest (ROI) (Fig. 3B, blue, Additional file 2: Appendix 1, Table 1). Other software packages include alternative building blocks to redefine areas, for example in CellProfiler we used “object processing” to “shrink” the object “cell” and identified a “tertiary object” by subtracting this modified area from the object “cytoplasm”, identifying a new object called “ROI” (Additional file 1: Fig. S1C, Additional file 2: Appendix 2). Within the defined ROI, we next used the Sec61β-mEmerald signal to detect ER tubules, and thereby form an ER network ‘mask’ (Fig. 3C, green; Additional file 1: Fig. S1C). By inverting the ER mask, we obtained the regions bounded by the ER tubules and cell perimeter (Fig. 3D, yellow and blue respectively; Additional file 1: Fig. S1C). As the regions between the ER and cell perimeter (Fig. 3D, blue) do not represent true ER polygons, these were excluded by filtering regions that overlapped with the cell perimeter. In this way, we were able to define the regions of the cytoplasm bounded by ER tubules as polygons (Fig. 3D, yellow; Additional file 1: Fig. S1C) that could be analysed by the software, and measurements such as mean area per cell (Fig. 3E) generated as a readout. This pipeline enables us to screen for chemical or genetic modifiers of the tubular SER network.

### Definition of perinuclear dense RER structure

**Fig. 4:**
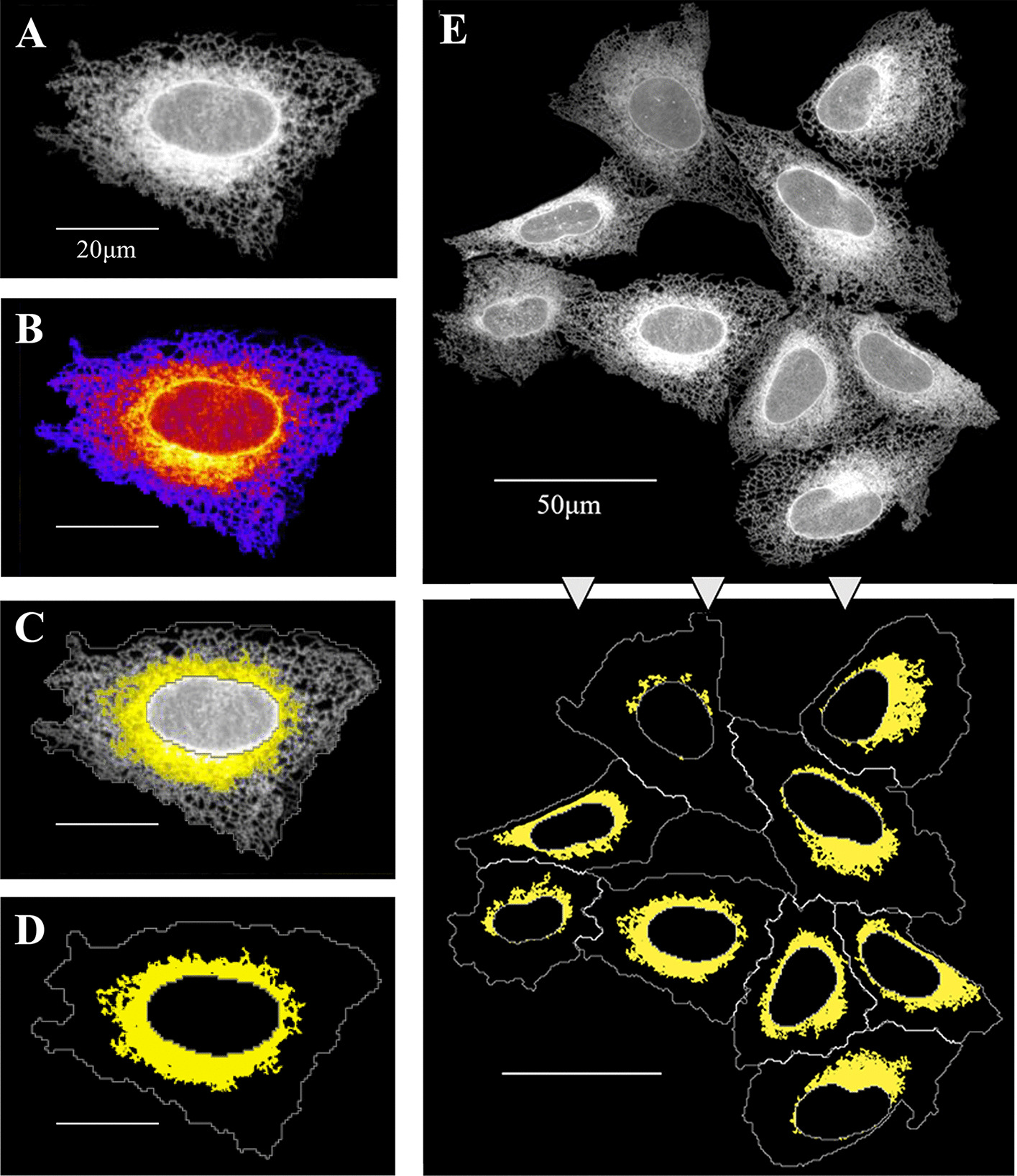
Definition of perinuclear dense RER structures. **A–D** Sequential key steps in the analysis shown on the same U-2 OS cell as in Fig. 2. Scale bars = 20 μm. **A** U-2 OS cell expressing the ER maker Sec61β-mEmerald (white). **B** Pseudo-coloured image based on Sec61β-mEmerald signal intensity, warm colours (red, orange, yellow) represent higher signal intensity, cold colours (pink, purple, blue) represent lower signal intensity. **C** Detection of dense ER (yellow area) after applying an intensity threshold to the Sec61β-mEmerald signal (white) within the cytoplasm (ROI). **D** Yellow region shows the surface of the cytoplasm, drawn in white, occupied by dense ER. **E** Steps A and D shown in a group of cells. Scale bar = 50 μm

The ribosome-studded RER is composed of a series of interconnected stacked sheets typically located in the perinuclear cell area [12, 48]. Although confocal microscopy cannot resolve ER substructures, we were able to clearly observe higher Sec61β-mEmerald intensity in the perinuclear cell area, relative to that seen in the periphery of the cell (Fig. 4A). The differential distribution in intensity of the ER marker becomes clearer on the pseudo-coloured image (Fig. 4B) with high intensity pixels coloured yellow or orange, and low intensity pixels coloured pink, purple or blue. The perinuclear region of high intensity Sec61β fluorescence likely corresponds to the area in which the RER resides, while the peripheral region corresponds to tubular SER, as previously discussed. Applying an intensity threshold, we generated a mask which broadly corresponds to the dense RER (Fig. 4C, yellow; Additional file 1: Fig. S1D). Once a mask of this region was created, we calculated the proportion of pixels of the cell area that overlap with it (Fig. 4D; Additional file 1: Fig. S1D). This analysis allowed us to calculate the proportion of RER within the cells (Fig. 4E), and determine how this sub compartment of the ER is altered upon treatment with chemical or genetic modifiers.

### Drug-induced ER reorganisation in U-2 OS cells

**Fig. 5:**
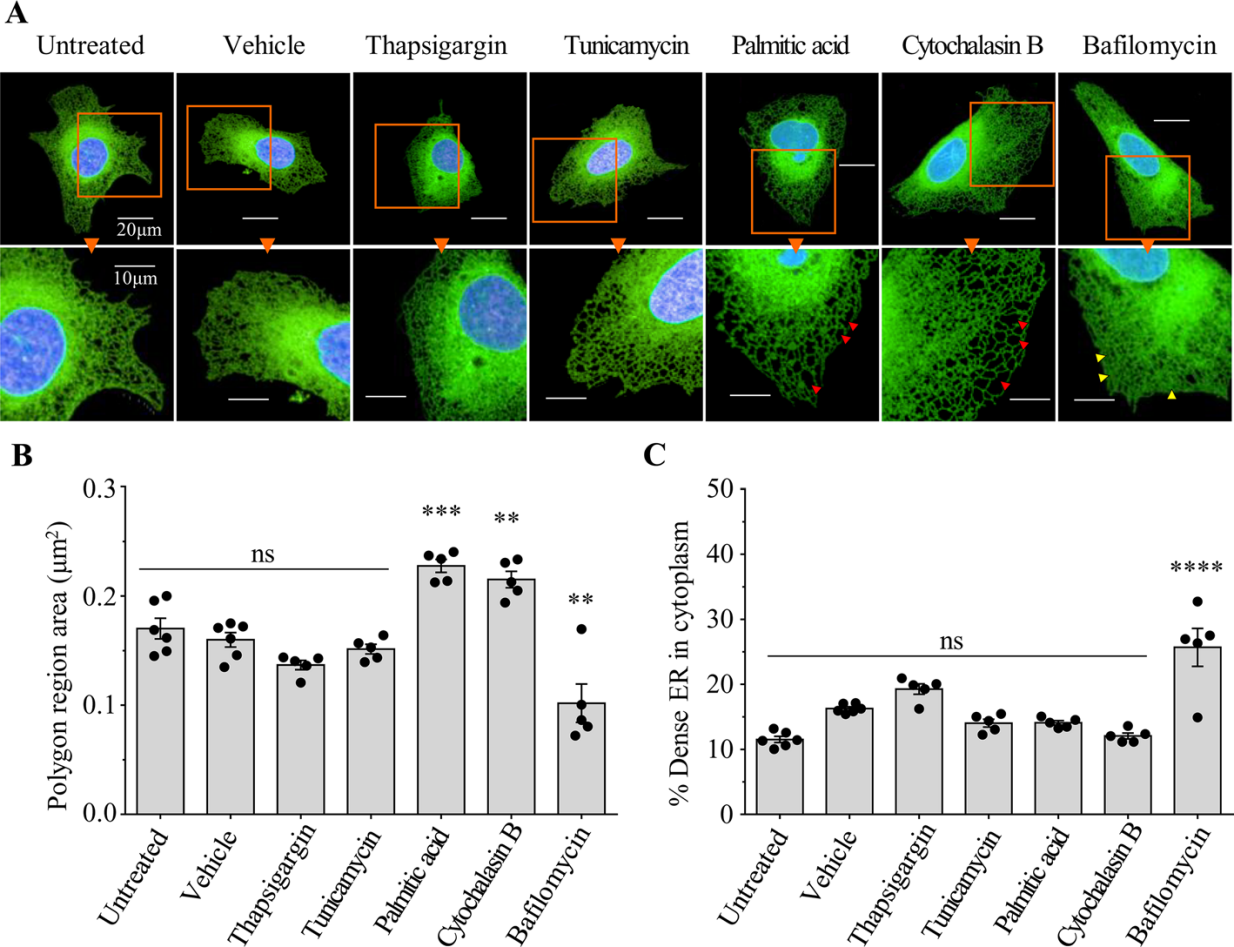
Drug-induced ER reorganisation in U-2 OS cells. **A** Representative images of ER (green) distribution in U-2 OS cells expressing Sec61β-mEmerald after treatment with vehicle (DMSO), Thapsigargin, Tunicamycin, Palmitic acid, Cytochalasin B and Bafilomycin A1. Polygon regions are seen to be enlarged (red arrowheads in enlarged images) or constricted (yellow arrowheads in enlarged images). Nucleus (blue). Scale bars = 20 μm top row images and 10 = μm lower row of enlarged images. **B** Quantification of ER polygon region average size. **C** Quantification of% of dense ER in cell cytoplasm. Data are expressed as mean ± SEM (n = 5 independent experiments comprising ≥ 50 cells each) and values significantly different from vehicle control were determined by one-way ANOVA and Tukey’s multiple comparisons test (**, *P* < 0.01; ***, *P* < 0.001; ****, *P* < 0.0001; ns, *P* > 0.05)

Several studies have reported alteration of ER organisation by different chemical compounds [32, 37, 49]. A limitation of all these studies however is that they employed qualitative analysis and did not enable an objective description of the changes observed. By contrast, the analysis pipeline that we have developed permits the unbiased quantification of changes to the ER network organisation in cultured cells upon drug treatment. We first applied our image analysis pipeline to the U-2 OS cells expressing Sec61β-mEmerald, and quantified the steady-state area of the SER polygons and the dense RER (Fig. 5). These values provided us with benchmark values for all subsequent experiments. Interestingly, when we repeated these experiments in another cell line, HeLa Kyoto, transiently expressing the same Sec61β-mEmerald protein, the values we obtained for SER and RER areas were highly comparable (Additional file 1: Fig. S2). This demonstrates that our image analysis strategy is not only restricted to making measurements from U-2 OS cells. To validate that our pipeline can also detect alterations in ER organisation, we treated U-2 OS cells expressing Sec61β-mEmerald with various drugs that have been reported to disrupt the ER. Following treatment with the saturated fatty acid palmitic acid (250 μM for 16 h) or the mycotoxin cytochalasin B (10 μM for 30 min), U-2 OS cells exhibited significantly larger polygon areas compared to untreated or DMSO vehicle controls (Fig. 5A, B) indicating that the SER network had become less dense. By contrast, treatment with the V-ATPase inhibitor bafilomycin A1 (50 nM for 16 h) resulted in significantly smaller SER polygons (Fig. 5A, B, Additional file 1: Fig. S3) and expansion of the dense RER (Fig. 5A, C, Additional file 1: Fig. S3) indicating an increase in the density of the ER network throughout the cell. Interestingly, drugs known to induce the ER stress response, thapsigargin (100 nM for 16 h) and tunicamycin (500 nM for 16 h) did not result in statistically significant alteration to the organisation of the ER network, at least at the resolution afforded by confocal fluorescence microscopy (Fig. 5).

### Analysis of ER distribution in U-2 OS cells using various ER markers

**Fig. 6:**
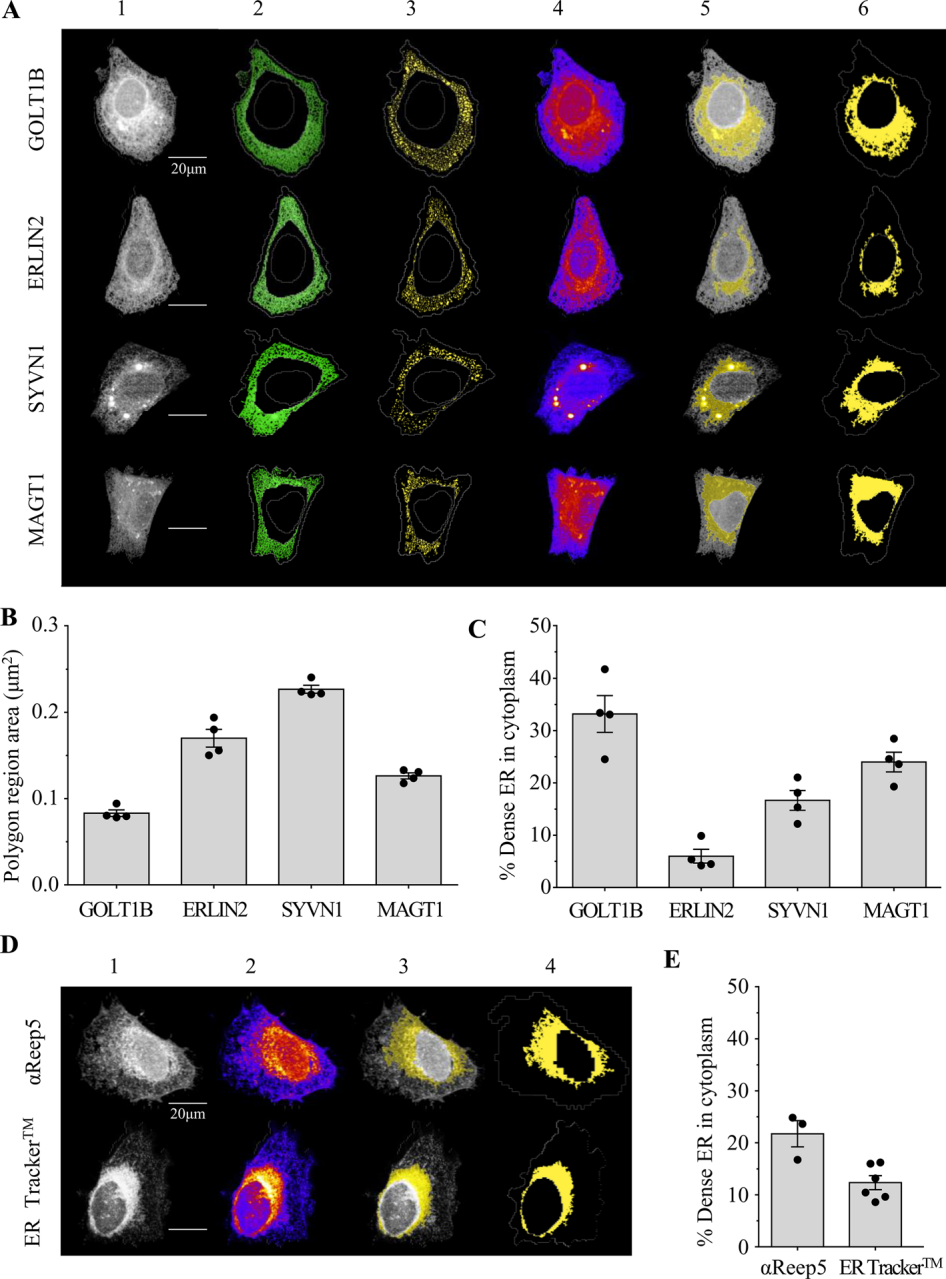
Analysis of ER distribution in U-2 OS cells using various ER markers. **A** Images of a single cell showing sequential analysis of SER polygon regions (columns 2 and 3) and dense RER (columns 4, 5 and 6) in U-2 OS cells expressing various constructs that label the ER (GOLT1B-YFP, ERIN2-YFP, SYVN1-YFP and MAGT1-YFP) (column 1). **B** Quantification of ER polygon region average size. **C** Quantification of % of dense ER in cell cytoplasm in U-2 OS cells expressing each ER-localising protein. **D** Representative images of U-2 OS cells showing sequential analysis of dense RER (columns 2, 3 and 4) labelled with anti-Reep5 antibody and ER Tracker™ (column 1) and quantification of % of dense ER in cell cytoplasm (**E**). Data are expressed as mean ± SEM (n = 3–4 independent experiments comprising ≥ 50 cells each). All scale bars = 20 μm

In order to validate that our analysis pipeline was compatible with alternative ER markers, we examined several over-expressed fluorescently-tagged ER-resident proteins, antibodies against ER residents, and the live-cell stain ER Tracker™ (Fig. 6). We selected four ER proteins, each with different functions, tagged with yellow fluorescent protein (YFP) from a library of expression constructs previously characterised in a large-scale subcellular localisation project [50, 51]. The conserved protein Golgi Transport 1B (GOLT1B) has been reported to localise to ER exit sites (ERES) and cis Golgi cisternae in yeast [52], and functions in mediating anterograde transport between the two organelles in rice endosperm cells [53]. Although the human orthologue has not been functionally described, a GOLT1B-YFP fusion protein has also been shown to localise to the ER in HeLa cells [51], and in our experiments in U-2 OS cells the GOLT1B-YFP protein localised to tubular structures with a high resemblance to SER tubules and dense perinuclear ER (Fig. 6A), suggesting that it is a *bona fide* ER marker. Similarly, we observed a high signal of the ER lipid-raft associated protein (ERLIN2) [54], the E3 ubiquitin-protein ligase synoviolin (SYVN1) [55], and a subunit of the oligosaccharyltransferase complex A (OST-A) Magnesium Transporter 1 (MAGT1) [56] YFP-tagged proteins on ER structures in U-2 OS cells (Fig. 6A). We adapted our previously described pipeline designed for Sec61β-mEmerald for each of these markers (Additional file 2: Appendix 1, Table 2) and successfully obtained metrics for ER polygon region area and % dense ‘RER’ in proportion to cell area (Fig. 6B, C).

Finally, we wanted to assess the applicability of our method beyond the use of over-expressed fluorescently-tagged proteins. To this end, we first used an immunofluorescence approach, applying our pipeline to immunolabelled ER-resident proteins. Several of the antibodies we tested only gave a low signal-to-noise ratio. We obtained the best results with an antibody against Reep5, although staining in the cell periphery was weak and did not show continuous tubules (Fig. 6D) precluding analysis of the polygon area. However, we were able to determine the proportion of dense ‘RER’ in relation to cell area using the anti-Reep5 antibody, as it provided a strong stain in the perinuclear area (Fig. 6D, E). Similarly, the frequently used ER Tracker dye failed to strongly label SER tubules but enabled analysis of dense ‘RER’ structures using our pipeline (Fig. 6D, E). Together, these results indicate that our analysis pipeline is compatible with a range of ER markers, while they also suggest caution in their choice, considering the advantages and limitations of each individual maker.

## Discussion

In this study we present an automated image analysis pipeline for the quantitative assessment of the ER using high-content fluorescence microscopy. Until now, the majority of studies reporting on ER morphology have been qualitative; i.e. describing the organelle as ‘normal’ or ‘abnormal’. Qualitative studies are limited to the visual detection of gross changes, such as may occur under high levels of stress or cell toxicity, but often fail to detect more subtle, yet physiologically relevant, changes. When quantitative analysis has been undertaken, it has been manually applied [57], an approach which is so labour intensive that it precludes its application to high-throughput screening as would be required to identify novel targets which could modify disease-associated ER alterations. Here we present two novel quantitative features that describe the ER, namely the SER tubule polygon area and the proportion of dense ‘RER’ in the cell. Together, these features enable key structures of the SER and RER to be reproducibly quantified in adherent mammalian cell lines.

Our experiments revealed that a cell line stably expressing the ER marker Sec61β-mEmerald provided the highest signal-to-noise ratio which facilitated our analysis. We were also able to demonstrate that our analysis pipeline can also be applied to other cell lines (HeLa Kyoto), as well as to cells stained with different classes of ER markers. Labelling of the ER using over-expression of tagged ER-resident proteins is a widely used strategy to assess ER organisation, however altered ER protein expression can itself disrupt ER organisation. For example, overexpression of the ER-shaping protein Lunapark leads to formation of unbranched SER tubules while its depletion leads to expansion of RER [40]. Consistent with this, our method detected changes to SER and RER organisation resulting from overexpression of the ER-resident proteins GOLT1B [51] and ERLIN2 [54]. Furthermore, our analysis determined that overexpression of the E3 ubiquitin ligase SYVN1 results in high SER polygon area in U-2 OS cells. This independently supports a recent study showing that overexpression of SYVN1 disrupts the SER network via ubiquitination-mediated inhibition of Atlastin-1 [55]. Therefore, our approach provides an opportunity to screen for proteins that regulate ER morphology and therefore potentially ER function.

The major strength of this automated approach is that it enables the rapid screening of genetic or chemical agents that can modify the ER as these may offer novel therapeutic targets for pathological conditions such as the motor neuron disorders HSPs, which are characterised by disrupted ER organisation. Here we conducted a pilot study on several drugs which have been linked to ER structure and function to validate the use of this pipeline to detect alterations in ER organisation. Thapsigargin and tunicamycin are highly efficient at inducing ER stress. Though ER stress and ER organisation have been linked in many studies, recent evidence suggests that treatment with either of these drugs alone is not sufficient to disrupt ER morphology [58, 59]. Likewise, here we report that treatment with thapsigargin or tunicamycin does not significantly alter tubular SER polygon area or the proportion of dense RER within U-2 OS cells. Lipid availability has been linked to ER morphology [25] and treatment with the saturated free fatty acid palmitate induces ER expansion and stress in several cell types [32, 60–63]. Here we show that treatment with a low concentration of palmitic acid increases the area of SER polygons. Similarly, actin filaments have a conserved role in maintenance of the ER network [35, 64, 65] and treatment with the actin‐depolymerising drug cytochalasin B alters ER content in developing oocytes (resulting in ER accumulation at the cortical region during oocyte activation) [37, 66]. Consistent with this, our pipeline was able to detect that brief treatment with cytochalasin B induces ER reorganisation with a significant increase in the area of SER polygons. ER structure and function are maintained by selective autophagic turnover, ER-phagy, impairment of which causes abnormal expansion of RER sheets [67]. Here we show that treatment with the autophagy inhibitor Bafilomycin A1 significantly reduces the area of SER polygons and increases the proportion of dense RER.

## Conclusions

Together, these findings support the validity of this automated pipeline for the analysis of ER features. This approach can be incorporated into large-scale image-based phenotypic screens, which could be used to identify genetic or chemical agents that modify the ER in normal or disease model cells.

## Methods

### Cell culture

Human bone osteosarcoma epithelial cells (U2-OS) stably expressing Sec61β-mEmerald [33], generously provided by Craig Blackstone, were cultured with 1 g/L glucose Dulbecco's modified Eagle's medium (DMEM) (Lonza LZBE12-707F) containing 10% heat inactivated foetal calf serum (Sigma-Aldrich F9665), 0.8% L-Glutamine (Thermo Scientific™ 25030024) and 10 μg/ml (or 0.1%) G-418 (Gibco 10131035), in a humidified incubator at 37 °C and 5% CO_2_. U-2 OS (ATCC® HTB-96™) and HeLa Kyoto (RRIC:CVCL_0042) cell lines used in transient transfection experiments were maintained in the same conditions but without antibiotic.

### Drug treatments

U-2 OS cells stably expressing Sec61β-mEmerald [33] were seeded at 4100 cells per well (equivalent to 1.3 × 10^4^ cells per cm^2^) in CellCarrier Ultra96-well optically clear plates (PerkinElmer). 16-h treatments started 24 h after seeding while 30-min treatments started at 39 h 30 min after seeding. Staggered treatments allowed synchronised fixation and staining of the entire plate. Cells were treated for 16 h with 0.5% DMSO (Sigma-Aldrich D2650), 250 μM Palmitic acid (Sigma-Aldrich P0500), 50 nM Bafilomycin A1 (B1793 Sigma-Aldrich), 100 nM Thapsigargin (Sigma-Aldrich T9033), and 500 nM Tunicamycin (Sigma-Aldrich T7765); and for 30 min with 10 μM Cytochalasin B (Sigma-Aldrich C6762).

### Transient transfection of ER-localising proteins

Non-transfected U-2 OS (RRID:CVCL_0042) and HeLa Kyoto (RRID:CVCL_1922) cells were seeded at 3500 cells per well (equivalent to 1.1 × 10^4^ cells per cm^2^) in CellCarrier Ultra96-well optically clear plates (PerkinElmer). 24 h after seeding, transfection complexes were prepared using 50 ng per well of various DNA constructs, using 0.13 μl of TransIT-X2 (Mirus MIR6003) in a final reaction volume of 20 μl OptiMEM. After 20 min incubation at room temperature, transfection reactions were added to the cells containing growth medium. The YFP-tagged constructs for transfection of the U-2 OS cells were obtained from a library of expression constructs described previously [50, 51]. For experiments not involving transient transfection, U-2 OS cells were instead stained with ER-Tracker™ (ThermoFisher E34251) or immunolabeled with anti-Reep5 antibodies (Proteintech 14643-1-AP) at the end of the culture time. HeLa Kyoto cells were transfected with Sec61β-mEmerald (Addgene plasmid #90992) [68]. The expression time was 24 h after addition of transfection complexes in all cases.

### Sample preparation for imaging

Following the various treatments, cells were incubated at 37 °C for 10 min with a pre-warmed solution of 10 μM Hoechst 33342 (Thermo Scientific™ 62249) and 1:1000 dilution of CellMask™ Deep Red plasma membrane stain (Invitrogen™ C10046) in DMEM medium to counterstain the nuclei and plasma membrane, respectively. In the case of ER Tracker™ stained cells, a 1 μM solution was used (ThermoFisher E34251). Next, cells were washed with phosphate-buffered saline (PBS) buffer, pH 7.4 and fixed with 4% (w/v) paraformaldehyde (PFA) for 15 min at room temperature. Subsequently, cells were washed 3 more times with PBS solution and kept in this solution for imaging. In the case of wells immunostained with anti-Reep5 antibodies, extra steps were followed, which comprised permeabilisation of the cell membrane with 0.01% Triton-X (Alfa Aeser A16046) in PBS for 10 min at room temperature; 30-min blocking with 0.5% FCS (Sigma-Aldrich F9665) in PBS; 1-h incubation with anti-Reep5 antibodies (Proteintech 14643-1-AP) in blocking solution; followed by 3 washes with PBS; 1-h incubation with anti-rabbit secondary Alexa 568 antibodies (ThermoFisher A11036) in blocking solution; and 3 final washes with PBS, after which cells were kept in this solution for imaging.

### Automated confocal imaging and analysis

Confocal images of 5 optical slices interspaced by 0.5 μm across 7 × 7 fields of view with a 10% overlay were acquired per well on an Opera Phenix High Content Screening System (PerkinElmer) using a 63 × /1.15 NA water-immersion objective with effective xy resolution of 0.28 μm. Images were analysed using Harmony® v.4.8 high-content analysis software (PerkinElmer), for pipeline see Additional file 2: Appendix 1. We used a computer powered by an Intel Xeon CPU at 3.60 GHz that contains 80 GB RAM, and runs a 64-bit operating system/Windows 10 Pro. Approximated computing time per 7 × 7 fields acquired per well was 10 min. A set of sample images (https://osf.io/nru7g/) was exported and used to recreate the analysis on CellProfiler v.3.1.8 [2], see Additional file 2: Appendix 2 for pipeline. Specific parameters require optimisation, as indicated Additional file 2: Appendixes 1 and 2, but once inputted the pipelines run to completion for the dataset provided. Cell segmentation was achieved using the CellMask™ channel. Throughout this paper, we quantified and analysed RER and SER morphology across populations of cells, analysing ≥ 50 cells per well. This ensured that inherent variability across a population of cultured cells was accounted for through the high numbers of cells analysed between replicate experiments (compare Fig. 5B,C with Additional file 1: Fig. S3). Raw data files containing per object and per cell results for experiments described within this work are available online at: https://osf.io/nru7g/.

## Supplementary Information

**Additional file 1: Fig. S1.** ER analysis pipeline in Cell Profiler. **Figure S2.** Pipeline for analysis of ER polygon regions and dense perinuclear ER applied to HeLa Kyoto (RRID:CVCL_1922) cells expressing Sec61β-mEmerald. **Figure S3.** Bafilomycin-induced ER reorganisation in U-2 OS cells.

**Additional file 2: Appendix 1.** Analysis sequence for ER analysis with Harmony: Table 1. Sequential building blocks in Harmony. Table 2. Pipeline settings adjusted for different experiments. **Appendix 2.** Analysis sequence for ER analysis with CellProfiler used in Fig. S1.

## Data Availability

The pipelines and sample raw images analysed during this study, in addition to sample raw data are available in the OSF repository, https://osf.io/nru7g/. The other example image data generated during this study are available from the corresponding author on request.

## Abbreviations

DMEM: Dulbecco's modified Eagle's medium
ER: Endoplasmic Reticulum
ERES: ER Exit Sites
ERLIN2: ER lipid-raft associated protein
GOLT1B: Golgi Transport 1B
HSPs: Hereditary Spastic Paraplegias
MAGT1: Magnesium Transporter 1
NE: Nuclear envelope
OST-A: Oligosaccharyltransferase complex A
RER: Rough ER
RFs: Replication factories
ROI: Region of interest
SER: Smooth ER
SYVN1: Synoviolin
U2-OS: Human bone osteosarcoma epithelial cells
