# Supplementary material for: A novel automated image analysis pipeline for quantifying morphological changes to the endoplasmic reticulum in cultured human cells
Source: BMC Bioinformatics. 2021 Sep 8;22:427. doi: 10.1186/s12859-021-04334-x (PMC8425006; doi:10.1186/s12859-021-04334-x)
Supplement: Supplementary file 1 — Additional file 1: Fig. S1. ER analysis pipeline in Cell Profiler. Figure S2. Pipeline for analysis of ER polygon regions and dense perinuclear ER applied to HeLa Kyoto (RRID:CVCL_1922) cells expressing Sec61β-mEmerald. Figure S3. Bafilomycin-induced ER reorganisation in U-2 OS cells. [file 12859_2021_4334_MOESM1_ESM.pdf]

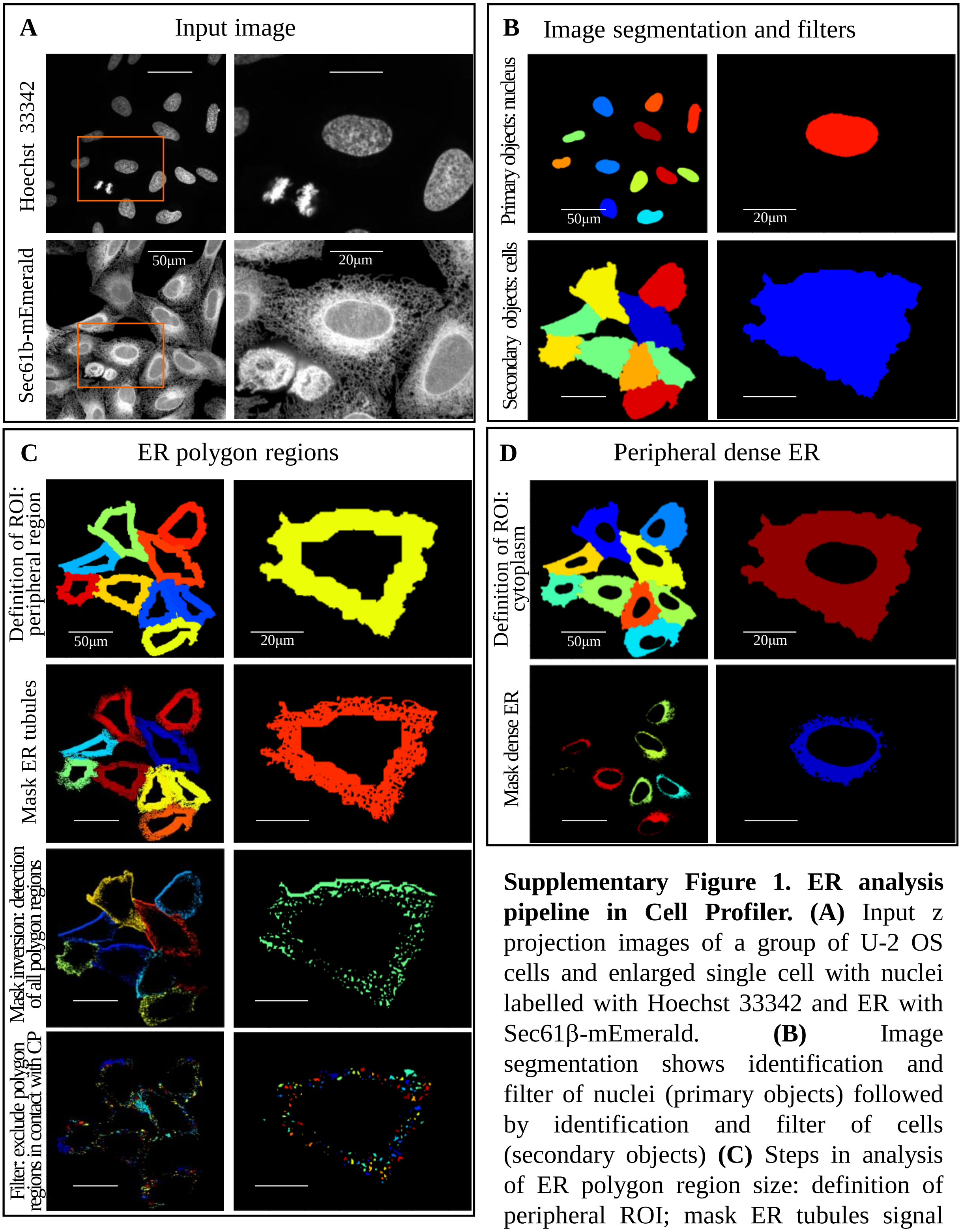

Supplementary Figure 1. ER analysis pipeline in Cell Profiler. (A) Input z projection images of a group of U-2 OS cells and enlarged single cell with nuclei labelled with Hoechst 33342 and ER with Sec61β-mEmerald. **(B)** Image segmentation shows identification and filter of nuclei (primary objects) followed by identification and filter of cells (secondary objects) **(C)** Steps in analysis of ER polygon region size: definition of peripheral ROI; mask ER tubules signal within ROI; inversion of mask; filter to exclude areas in contact with CP (cell perimeter) keeping polygon regions to measure. **(D)** Steps in analysis of dense ER: definition cytoplasm as ROI; mask dense ER signal within ROI. All scale bars in the group of cells are 50µm; and in the enlarged single cell 20µm.

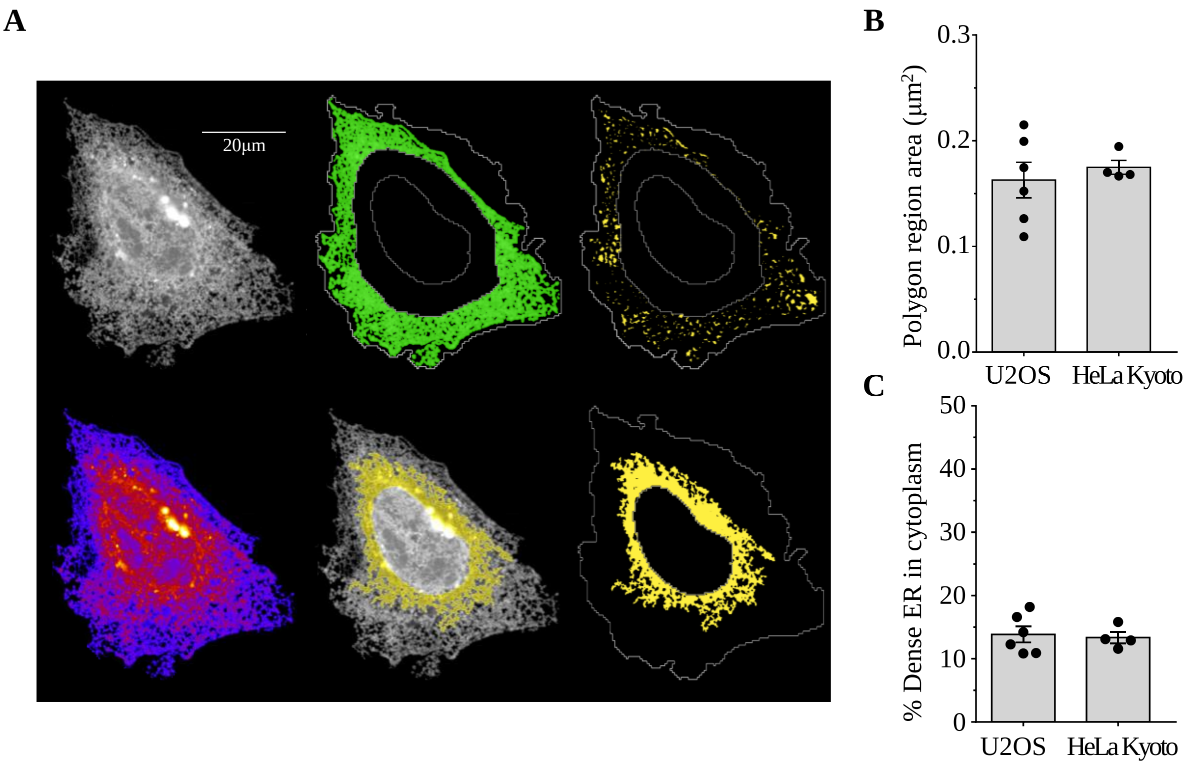

Supplementary Figure 2. Pipeline for analysis of ER polygon regions and dense perinuclear ER applied to HeLa Kyoto (RRID:CVCL_1922) cells expressing Sec61β-mEmerald. (A) Images of key steps of the ER analysis described in Figures 3 and 4 showing analysis of ER polygon region area and dense ER in HeLa Kyoto cells expressing Sec61β-mEmerald. Scale bar 20μm **(B)** Quantification of polygon region size **(B)** and % dense ER in cytoplasm **(C)** in HeLa cells compared to U-2 OS cells expressing the same ER marker. Data are expressed as mean ± SEM (n ≥50 cells from 4-6 independent experiments).

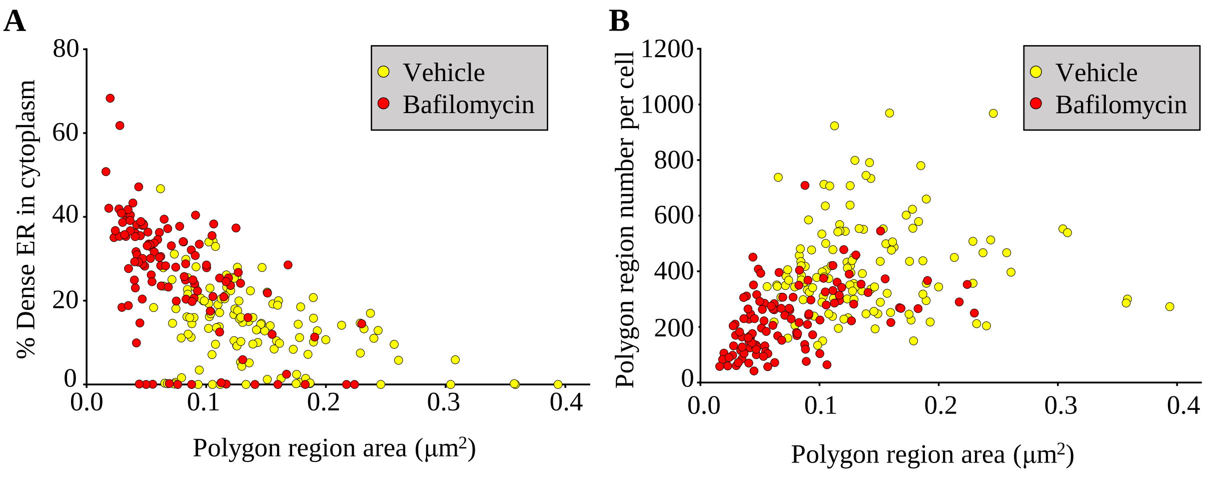

C

	Polygon region area (μm^2)	% Dense ER in cytoplasm	Polygon number per cell	Cell number
**Vehicle (DMSO)** *Mean ± SEM*	0.14070 ± 0.005615	14.08 ± 0.8284	420.7 ± 15.39	N=125
**Bafilomycin** *Mean ± SEM*	0.07552 ± 0.004183	26.97 ± 1.2440	230.4 ± 10.83	N=118
Two-tailed P-value	***0.0005*** ***	***<0.0001*** ****	***<0.0001*** ****	

Supplementary Figure 3. Bafilomycin-induced ER reorganisation in U-2 OS cells. Individual cell analysis of a single experiment of the five shown in Fig. 5. comparing vehicle (DMSO) and bafilomycin treated cells. Scatter plots comparing **(A)** cell mean polygon region area (μm^2) and % of dense ER in cytoplasm; **(B)** cell mean polygon region area (μm^2) and number of polygon regions per cell, show cells treated with vehicle (DMSO) in yellow and cells treated with bafilomycin in red. **(C)** Table summarising data shown in A and B.
