# Supplementary material for: A novel automated image analysis pipeline for quantifying morphological changes to the endoplasmic reticulum in cultured human cells
Source: BMC Bioinformatics. 2021 Sep 8;22:427. doi: 10.1186/s12859-021-04334-x (PMC8425006; doi:10.1186/s12859-021-04334-x)
Supplement: Supplementary file 2 — Additional file 2: Appendix 1. Analysis sequence for ER analysis with Harmony: Table 1. Sequential building blocks in Harmony. Table 2. Pipeline settings adjusted for different experiments. Appendix 2. Analysis sequence for ER analysis with CellProfiler used in Fig. S1. [file 12859_2021_4334_MOESM2_ESM.pdf]

Appendix 1. Analysis sequence for ER analysis with Harmony

Appendix 1. **Table 1. Sequential building blocks in Harmony**

**Input image settings**	**Create Global Image:** Min. Global Binning : Dynamic<br>**Flatfield Correction :** Advanced<br>**Stack Processing :** Maximum Projection

Underlined parameters and text defined by user; * thresholds used in each experiment detailed in Table 2

Building block	Input	Method	Output
**1.**Filter image	**Channel :** HOECHST 33342 (global)	**Method :** Smoothing<br>Filter : Gaussian<br>Width : 1 px	**Output Image :** "HOECHST 33342 (global) gaussian"
**2.** Find nuclei	**Channel:** "HOECHST 33342 (global) gaussian"<br>**ROI :** Imaged Area (global)<br>**ROI Region :** Imaged Area	**Method :** B<br>Common Threshold : 0.5<br>Area : > 30 µm²<br>Splitting Coefficient : 7.5<br>Individual Threshold : 0.2<br>Contrast : > 0.1	**Population :** "Nuclei global"
**3.** Calculate image		**Method :** By Formula<br>Formula : A+B+C<br>Channel A : HOECHST33342 (global)<br>Channel B : CellMask (global)<br>Channel C : ER marker (global)<br>Negative Values : Set to Zero<br>Undefined Values : Set to Local	**Output Image :** "Calculated 3 channels global"
**4.** Filter image	**Channel :** "Calculated 3 channels global"	**Method :** Smoothing<br>Filter : Gaussian<br>Width : 1 px	**Output Image :** "Calculated 3 channels global gaussian"
**5.** Filter image	**Channel :** "Calculated 3 channels global gaussian"	**Method :** Sliding Parabola<br>Curvature : 1	**Output Image :** "Calculated 3 channels global gaussian sliding parabola"
**6.** Filter image	**Channel :** CellMask (global)	**Method :** Texture SER<br>Filter : SER Edge<br>Scale : 1 px<br>Normalization by : Kernel	**Output Image :** "CellMask edge"
**7.** Find cytoplasm	**Channel :** "Calculated 3 channels global gaussian sliding parabola"<br>**Nuclei : "**Nuclei global"	**Method :** F<br>Membrane Channel : "CellMask edge"<br>Individual Threshold : 0.01<br>Restrictive Region : Imaged Area	**Output regions:**<br>Region: Cell<br>Region: Cytoplasm<br>Region: Nucleus<br>Region: Plasma membrane
**8.** Calculate morphology properties	**Population : "**Nuclei global"<br>**Region :** Nucleus	**Method :** Standard<br>Area<br>Roundness	**Property Prefix :** "Nucleus morphology properties"

**9.** Calculate intensity properties	**Channel :** ER marker (global)<br>**Population : "**Nuclei global"<br>**Region :** Cell	**Method :** Standard Mean	**Property Prefix :** "Cell ER marker intensity properties"
**10.** Calculate intensity properties	**Channel :** HOECHST 33342 (global)<br>**Population : "**Nuclei global"<br>**Region :** Nucleus	**Method :** Standard Mean	**Property Prefix :** "Nucleus intensity properties"
**11.** Select population	**Population : "**Nuclei global"	**Method :** Filter by Property<br>Cell morphology Area (µm²): > 700<br>Cell morphology Area (µm²): < 4500<br>Cell morphology Ratio Width to Length: > 0.3<br>Nucleus morphology Area (µm²): > 100<br>Nucleus morphology Area (µm²): < 700<br>Nucleus morphology Roundness : > 0.8<br>Cell ER marker intensity Mean : > A *** Table 2**<br>Nucleus intensity Mean : < 3000	**Population :** "Selected cells"
**12.** Calculate image		**Method :** By Formula<br>Formula : A+B+C<br>Channel A : CellMask<br>Channel B : HOECHST 33342<br>Channel C : ER marker<br>Negative Values : Set to Zero<br>Undefined Values : Set to Local Average	**Output Image :** "Calculated 3 Channels single field"
**13.** Filter image	**Channel : "**Calculated 3 Channels"	**Method :** Smoothing<br>Filter : Gaussian<br>Width : 3 px	**Output Image :** "Calculated 3 channels single field gaussian"
**14.** Find image region	**Channel :** "Calculated 3 channels single field gaussian"<br>**ROI : "**Selected cells"<br>**ROI Region :** Cell	**Method :** Absolute Threshold<br>Lowest Intensity : ≥ 1<br>Highest Intensity : ≤ INF<br>Split into Objects<br>Area : > 0 µm²	**Population :** "(cell)"<br>**Output Region :** "(cell)"
**15.** Select population	**Population : "**(cell)"	**Method :** Common Filters<br>Remove Border Objects<br>Region : "(cell)"	**Population :** "CELL"
**16.** Find image region	**Channel :** "Calculated 3 channels single field gaussian"<br>**ROI : "**Selected cells"<br>**ROI Region :** Cytoplasm	**Method :** Absolute Threshold<br>Lowest Intensity : ≥ 1<br>Highest Intensity : ≤ INF<br>Split into Objects<br>Area : > 0 µm²	**Population :** "(cytoplasm)"<br>**Output Region :** "(cytoplasm)"
**17.** Select population	**Population :** "(cytoplasm)"	**Method :** Common Filters<br>Remove Border Objects<br>Region : "(cytoplasm)"	**Population :** "CYTOPLASM"

**18.** Find image region	**Channel :** "Calculated 3 channels single field gaussian"<br>**ROI : "**Selected cells"<br>**ROI Region :** Nucleus	**Method :** Absolute Threshold<br>Lowest Intensity : ≥ 1<br>Highest Intensity : ≤ INF<br>Split into Objects<br>Area : > 0 µm²	**Population :** "NUCLEUS"<br>**Output Region** : "NUCLEUS"
**19.** Find image region	**Channel :** "Calculated 3 channels single field gaussian"<br>**ROI : "**Selected cells"<br>**ROI Region :** Plasma membrane	**Method :** Absolute Threshold<br>Lowest Intensity : ≥ 1<br>Highest Intensity : ≤ INF<br>Split into Objects<br>Area : > 0 µm²	**Population :** "PLASMA MEMBRANE"<br>**Output Region :** "PLASMA MEMBRANE"
**20.** Select cell region	**Population :** "Selected cells"	**Method : Method :** Resize Region [%]<br>Region Type : Ring Region<br>Outer Border : 0 %<br>Inner Border : 25 %	**Output Region :** "Cytoplasmic peripheral region"
**21.** Find image region	**Channel :** "Calculated 3 channels single field gaussian"<br>**ROI :** Selected cells<br>**ROI Region :** "Cytoplasmic peripheral region"	**Method :** Absolute Threshold<br>Lowest Intensity : ≥ 1<br>Highest Intensity : ≤ INF<br>Split into Objects<br>Area : > 0 µm²	**Population :** "(cytoplasmic peripheral region)"<br>**Output Region :** "(cytoplasmic peripheral region)"
**22.** Select population	**Population : "**(cytoplasmic peripheral region)"	**Method :** Common Filters<br>Remove Border Objects<br>Region : "(cytoplasmic peripheral region)"	**Population :** "CYTOPLASMIC PERIPHERAL REGION"
**23.** Filter image	**Channel :** ER marker	**Method :** Smoothing<br>Filter : Gaussian<br>Width : 0.5 px	**Output Image :** "ER marker gaussian"
**24.** Filter image	**Channel :** "ER marker"	**Method :** Sliding Parabola<br>Curvature : 10	**Output Image :** "ER marker gaussian sliding parabola"
**25.** Find image region	**Channel :** "ER marker gaussian sliding parabola"<br>**ROI :** "CYTOPLASMIC PERIPHERAL REGION"<br>**ROI Region :** "(cytoplasmic peripheral region)"	**Method :** Absolute Threshold<br>Lowest Intensity : ≥ B ***Table 2**<br>Highest Intensity : ≤ INF<br>Split into Objects<br>Area : > 6 µm²	**Population :** "ER in peripheral cytoplasm"<br>**Output Region :** "ER in peripheral cytoplasm"
**26.** Select region	**Population :** "CYTOPLASMIC PERIPHERAL REGION"<br>**Region :**"(cytoplasmic peripheral region)"	**Method :** Restrict by Mask<br>Population : : "ER in peripheral cytoplasm"<br>Mask Region : "ER in peripheral cytoplasm"<br>Use Inverted Mask	**Output Region :** "All polygonal regions"

**27.** Find image region	**Channel :** "Calculated 3 channels single field gaussian" **ROI :** "CYTOPLASMIC PERIPHERAL REGION" **ROI Region :** "All polygonal regions"	**Method :** Absolute Threshold<br>Lowest Intensity : ≥ 1<br>Highest Intensity : ≤ INF<br>Split into Objects<br>Area : > 0 µm²	**Population :** "All polygonal regions" **Output Region :** "All polygonal regions"
**28.** Calculate position properties	**Population :** "All polygonal regions" **Region :** "All polygonal regions"	**Method :** Cross Population<br>Population B : "PLASMA MEMBRANE"<br>Region B : "PLASMA MEMBRANE"<br>Overlap	**Property Prefix :** "All polygon regions and plasma membrane"
**29.** Select population	**Population :** "All polygonal regions"	**Method :** Filter by Property<br>"All polygon regions and plasma membrane" overlap (%): ≤ 0.1	**Population :** "POLYGON REGIONS"
**30.** Calculate morphology properties	**Population :** "POLYGON REGIONS"	**Method :** Standard<br>Area	**Property Prefix :** "POLYGON REGION morphology properties"
**31.** Find image region	**Channel :** ER marker **ROI :** "CYTOPLASM" **ROI Region :** "(cytoplasm)"	**Method :** Absolute Threshold<br>Lowest Intensity : ≥ C ***Table 2**<br>Highest Intensity : ≤ INF<br>Split into Objects<br>Area : > 0 µm²	**Population :** "(Dense ER)" **Output Region :** "(Dense ER)"
**32.** Calculate position properties	**Population : "**(Dense ER)" **Region : "**(Dense ER)"	**Method :** Cross Population<br>Population B : "NUCLEUS"<br>Region B : "NUCLEUS"<br>Nearest Object Distance	**Property Prefix :** "Dense ER and nucleus"
**33.** Select population	**Population : "**(Dense ER)"	**Method :** Filter by Property<br>"Dense ER and nucleus" Nearest Object Distance (µm) : ≤ 0.001	**Population :** "Dense ER"
**34.** Calculate position properties	**Population : "**CELL" **Region : "**(cell)"	**Method :** Cross Population<br>Population B : "Dense ER"<br>Region B : "(Dense ER)"<br>Overlap	**Property Prefix :** "CELL and Dense ER"
**35.a.** Export object results	**Population :** "CELL"	**Method :** Object results<br>➢ "Dense ER" Overlap (%)	
	**Population :** "POLYGON REGIONS"	**Method :** Object results<br>➢ "POLYGON REGION morphology properties" Area	
**35.b.** Export well results	**Population :** "CELL"	**Method :** Well results<br>➢ "Dense ER" Overlap (%) - Mean + Stdev	
	**Population :** "POLYGON REGIONS"	**Method :** Well results<br>➢ "POLYGON REGION morphology properties" Area- Mean + Stdev Mean<br>➢ Number of objects	

Appendix 1. **Table 2. Pipeline settings adjusted for different experiments**

	Thresholds *		
**Experiment /** sample preparation	A (Used in building block 11)	B (Used in building block 25)	C (Used in building block 31)
**Fig. 3, 4 and 5 /** U2-OS stably expressing Sec61β-mEmerald	*600*	*15*	*5,500*
**Fig. 6 A, B and C /** U-2 OS transiently expressing GOLT1B-YFP	*500*	*12*	*2,200*
**Fig. 6 A, B and C /** U-2 OS transiently expressing ERLIN2-YFP	*220*	*10*	*750*
**Fig. 6 A, B and C** U-2 OS transiently expressing SYVN1-YFP	*280*	*7*	*500*
**Fig. 6 A, B and C /** U-2 OS transiently expressing MAGT1-YFP	*200*	*15*	*500*
**Fig. 6 D and E /** U-2 OS immunolabelling of Reep5	*250*	-	*450*
**Fig. 6 D and E /** U-2 OS staining with ER-tracker	*150*	-	*360*
**Supplementary Fig. 2 /** HeLa Kyoto transiently expressing Sec61β-mEmerald	*1,000*	*15*	*10,000*

Appendix 2. Analysis sequence for ER analysis with CellProfiler used in Supplementary Fig. 1

Underlined parameters and text defined by user

Building block	Input & Method	Output
**Names And Types**	**Assign a name to images matching rules**<br>1. File Does Contain "HOECHST";<br>2. File Does Contain "CellMask";<br>3. File Does Contain "EGFP";<br>**Image type:** Grayscale image; **Intensity range from:** Image metadata.	**Output images:**<br>1.Name: "HOECHST"<br>2.Name: "CellMask"<br>3.Name: "ER"
**1.** Smooth	**Input:** HOECHST<br>Gaussian Filter<br>Calculate artifact diameter automatically	**Output:**<br>"HOECHST_ Gaussian"
**2.** Identify Primary Object	**Input:** "HOECHST_Gaussian"<br>Min 200 Max 400<br>Discard objects outside diameter rage: Y<br>Discard objects border image: Y	**Output:** "Nucleus"
**3.** Smooth	**Input:** CellMask<br>Gaussian Filter<br>Typical artifact diameter: 20	**Output:**<br>"CellMask_ Gaussian"
**4.** Enhance Edges	**Input:** "CellMask_Gaussian"<br>Edge-finding method: Kirsch	**Output:**<br>"CellMask_Edges"
**5.** Rescale Intensity	**Input:** "CellMask_Gaussian"<br>Method: Stretch each image to use full intensity range	**Output:**<br>"CellMask_ RescaleIntensity"
**6.** Image Math	Operation: Multiply<br>**Input:** Image 1: "CellMask_Edges" ; multiply by 20<br>Image 2: "CellMask_Edges"; multiply by 20<br>(Raise to the power of 1 Multiply by 1 Add 0)<br>Values less than 0 equal to 0 Y<br>Values greater than 1 equal to 1 Y<br>Ignore image masks N	**Output:**<br>"CellMask_Edges_ Calculated"
**7.** Correct Illumination apply	**Input:** "CellMask_ RescaleIntensity"<br>Illumination function: "CellMask_Edges_ Calculated"<br>How illumination function is applied: Substract	**Output:**<br>"CellMask_ Improved"
**8.** Identify Secondary Objects	**Input:** Image "CellMask_ Improved"<br>Objects: "Nucleus"<br>Method: Watershed Gradient<br>Threshold strategy: Adaptative, Otsu, Two-classes<br>Smoothing scale: 3; Correction factor: 1<br>Lower and upper bounds: 0.1 & 1<br>Fill holes Y; Discard objects touching border: Y;<br>Discard primary associated objects: N	**Output:**<br>"Cells_Without_ Filter"
**9.** Measure Object Intensity	**Input Image to measure:** ER<br>**Input Objects to measure:** "Cells_Without_ Filter"	

**10.** Filter Objects	**Input objects:** "Cells_Without_ Filter"<br>Mode: Measurements; Method: Limits; Mean Intensity; "ER"<br>Minimum measurement value: 0.018	**Output:** "Cells"
**11.** Identify Tertiary Object	**Input:** Larger object: "Cells"<br>Smaller Object: "Nucleus"<br>Shrink prior subtraction: N	**Output:** "Cytoplasm"
**12.** Expand or Shrink Objects	**Input:** "Cells"<br>Shrink by Number of pixels: 75<br>Shrink to a single point: N	**Output:** "Shrunken_cell"
**13.** Identify Tertiary Object	**Input:** Larger object: "Cytoplasm"<br>Smaller Object: "Shrunken_cell"<br>Shrink prior subtraction: N	**Output:** "ROI"
**14.** Smooth	**Input:** ER<br>Gaussian Filter<br>Calculate artifact diameter value: 0.5	**Output:** "ER_Gaussian"
**15.** Reduce Noise	**Input:** "ER_Gaussian"<br>Gaussian Filter<br>Size: 1<br>Distance: 1<br>Cut-off distance: 0.1	**Output:** "ER _ReducedNoise"
**16.** Enhance or Suppress Features	**Input:** "ER _ReducedNoise"<br>Operation: Enhance<br>Feature type: Neurites<br>Method: Line Structures<br>Feature size: 5	**Output:** "ER_Enhanced_ Tubules"
**17.** Threshold	**Input:** "ER_Enhanced_Tubules"<br>Strategy: Global<br>Method: Manual<br>Threshold: 0.0175	**Output:** "Threshold_ ER_Tubules"
**18.** Mask Objects	**Object to be Masked:** "ROI"<br>Masking Using Binary Image: "Threshold_ER_Tubules"<br>Invert: N<br>Keep Overlapping Region<br>Renumber	**Output:** "ROI_ER_Tubules"
**19.** Mask Objects	**Object to be Masked:** "ROI"<br>Masking Using Binary Image: "Threshold_ER_Tubules"<br>Invert: Y<br>Keep Overlapping Region<br>Renumber	**Output:** "ROI_All_ Polygon_Regions"
**20.** Split or Merge Objects	**Input:** "ROI_All_Polygon_Regions"<br>Split	**Output:** "ROI_ Individual_All_ Polygon_Regions"
**21.** Expand or Shrink Objects	**Input:** "Cells"<br>Shrink by Number of pixels: 10<br>Shrink to a single point: N	**Output:** "Shrunken_cell_2"
**22.** Identify Tertiary Object	**Input:** Larger object: "Cell"<br>Smaller Object: "Shrunken_cell_2"<br>Shrink prior subtraction: N	**Output:** "Cell_Perimeter"

**23.** Mask Objects	**Object to be Masked:** "ROI_ Individual_All_ Polygon_Regions"<br>Masking Using Object: "Cell_Perimeter"<br>Invert: Y<br>Remove Depending on Overlap<br>Fraction of Object that Must Overlap: 1<br>Renumber	**Output:** "Polygon_Regions"
**24.** Measure Object Size and Shape	**Input: "**Polygon_Regions"<br>Zernike Features: N	
**25.** Relate Objects	**Parent:** "Cells"<br>Child: "Polygon_regions"<br>Calculate per-parent mean: Y	**Output: "**Polygon_ Regions_in_Cell"
**26.** Threshold	**Input: "**ER"<br>Strategy: Global<br>Method: Manual<br>Threshold: 0.15	**Output image:** "Threshold_Dense_ ER"
**27.** Mask Objects	**Object to be Masked: "**Cytoplasm"<br>Masking Using Binary Image: "Threshold_Dense_ER"<br>Invert: N<br>Keep Overlapping Region<br>Renumber	**Output object:** "Threshold_Dense_ ER"
**28.** Split or Merge objects	**Input:** "Threshold_Dense_ER"<br>Split	**Output object:** "Split_Dense_ER"
**29.** Expand or Shrink Objects	**Input:** "Nucleus"<br>Expand by Number of pixels 2	**Output image:** "Expanded_ Nucleus"
**30.** Mask Objects	**Object to be Masked: "**Split_Dense_ER"<br>Masking Using Objects: "Expanded_Nucleus"<br>Invert: N<br>Keep & Renumber	**Output:** "Perinuclear_Dense _ER"
**31.** Relate Objects	**Parent:** "Cells"<br>Child: "Perinuclear_Dense_ER"<br>Calculate per-parent mean: Y	**Output: "**Dense_ER_ in_Cell"
**32.** Split or Merge objects	**Input: "**Dense_ER_ in_Cell"<br>Merge<br>Method: Per-parent; Disconnected<br>Parent: "Cell"	**Output object:** "Dense_ER"
**33.** Calculate Math	Operation: Divide; **Input**:<br>• Numerator Type: Object; "Dense_ER"<br>Measurement: Area; (Multiply by 1; Raise to the power of 1)<br>• Denominator Type: Object; "Cell"<br>Measurement: Area; (Multiply by 1; Raise to the power of 1)<br>Take log10 of Result: N<br>Multiply the Result by: 100<br>(Raise to the power of 1 Add 0)<br>Constrain result to lower or upper bound: N	**Output object:** "Percentage_Dense ER_in_Cell"

**33.** Measure Object Size and Shape	**Input:** “Dense_ER ”<br>Zernike Features: N	
**34.** Export to Spread Sheet	**Selected Measurements:**<br>Object: “Cells”: Area<br>Children; “Polygon_Regions”; Count<br>Mean; “Polygon_Regions”; Area<br>Math; “Percentage_DenseER_in_Cell”<br>Object: Dense_ER; Area<br>Object: Polygon_regions; Area & Parent	**Data to export:**<br>“Cells”<br>“Dense_ER”<br>(Combine with previous measurement Y)<br>“Polygon_ Regions”<br>(Combine with previous measurement N)
